# Regulation of cerebrospinal fluid production by caffeine consumption

**DOI:** 10.1186/1471-2202-10-110

**Published:** 2009-09-03

**Authors:** Myoung-Eun Han, Hak-Jin Kim, Young-Suk Lee, Dong-Hyun Kim, Joo-Taek Choi, Chul-Sik Pan, Sik Yoon, Sun-Yong Baek, Bong-Seon Kim, Jae-Bong Kim, Sae-Ock Oh

**Affiliations:** 1 Department of Anatomy, School of Medicine, Pusan National University, Busan, South Korea; 2 Medical Research Center for Ischemic Tissue Regeneration, Pusan National University, Busan, South Korea; 3 Department of Radiology, School of Medicine, Pusan National University, Busan, South Korea; 4 Department of Biomedical Engineering, School of Medicine, Pusan National University, Busan, South Korea

## Abstract

**Background:**

Caffeine is the most commonly consumed psycho-stimulant in the world. The effects of caffeine on the body have been extensively studied; however, its effect on the structure of the brain has not been investigated to date.

**Results:**

In the present study we found that the long-term consumption of caffeine can induce ventriculomegaly; this was observed in 40% of the study rats. In the caffeine-treated rats with ventriculomegaly, there was increased production of CSF, associated with the increased expression of Na^+^, K^+^-ATPase and increased cerebral blood flow (CBF). In contrast to the chronic effects, acute treatment with caffeine decreased the production of CSF, suggesting 'effect inversion' associated with caffeine, which was mediated by increased expression of the A_1 _adenosine receptor, in the choroid plexus of rats chronically treated with caffeine. The involvement of the A_1 _adenosine receptor in the effect inversion of caffeine was further supported by the induction of ventriculomegaly and Na^+^, K^+^-ATPase, in A_1 _agonist-treated rats.

**Conclusion:**

The results of this study show that long-term consumption of caffeine can induce ventriculomegaly, which is mediated in part by increased production of CSF. Moreover, we also showed that adenosine receptor signaling can regulate the production of CSF by controlling the expression of Na^+^, K^+^-ATPase and CBF.

## Background

Methylxanthine caffeine is present in many common beverages, and is widely consumed worldwide [1,4]. Caffeine consumption has been estimated to be 76 mg per person per day worldwide, as high as 238 mg per person per day in the United States and Canada, and more than 400 mg per person per day in Sweden and Finland [5,6]. Caffeine is absorbed rapidly after oral administration and distributed to various organs and tissues. In the liver, caffeine is metabolized to dimethyl- and monomethylxanthines, dimethyl and monomethyl uric acids, trimethyl- and dimethylallantoin, and uracil derivatives. Some metabolites of caffeine including 1,3-dimethylxanthine (theophylline) and 1,7-dimethylxanthine (paraxanthine) have pharmacological activity similar to caffeine [4]. The half-life of caffeine is ~5 hours in humans and ~1 hour in rats [4,7].

The main mechanism of action of caffeine is by antagonism of the adenosine receptors [4]. Among four adenosine receptors (A_1_, A_2A_, A_2B_, A_3_), the A_1 _and A_2A _receptors are high affinity receptors and are abundantly expressed. These receptors can be activated at low basal adenosine concentrations. Thus, by blocking the action of endogenous ligands, at these receptors, caffeine can exert its biological effects. A_2B _and A_3 _adenosine receptors require higher concentrations of adenosine for activation. Adenosine A_1 _receptors have been associated with caffeine's effect on neurotransmitter release. A_2A _receptor knockout mice respond less or not at all to caffeine on locomotion and wakefulness testing [8,10].

Interestingly, short- and long-term treatment with caffeine has different effects. Short-term treatment with caffeine decreases the threshold for convulsions [11,12]. By contrast, long-term treatment with caffeine increases the threshold for convulsions [13,14]. Moreover, short-term treatment with caffeine worsens ischemia-induced damage [15], whereas, long-term treatment with caffeine reduces such damage [16,17]. Despite these different effects of long-term and short-term treatment, the underlying mechanism associated with the long-term effects of caffeine has not been well characterized.

While studying the underlying mechanisms of caffeine-induced impairment in learning and memory, we noted frequent enlargement of the ventricles in caffeine-treated rats. Therefore, this study was undertaken to investigate the underlying mechanisms of caffeine-induced ventriculomegaly. We found that overproduction of CSF in caffeine-treated rats causes ventriculomegaly.

## Methods

### Animals

Male Sprague-Dawley rats (body weight 280 - 320 g, 7-10 weeks old) were caged in an air-conditioned room maintained at 22 ± 2°C, relative humidity 50 ± 10%, with a 12/12 h light/dark cycle. Animals had free access to tap water and were fed a conventional rat chow diet. They were acclimated for 1 week prior to beginning the study. Procedures related to animal care were in accord with the guidelines of the 'Guide for the Care and Use of Laboratory Animals' [18]. Caffeine (0.3 or 0.6 g/L, Sigma-Aldrich, USA) was added to the drinking water. The Pusan National University Institutional Animal Care and Use Committee (PNUIACUC) approved the experimental procedures.

### Examination of brain structure

To examine the brain structure in the rats, magnetic resonance imaging (MRI) (4.7 T Bruker Biospin, Germany or 1.5 T Siemens, Germany) and H&E staining were used. T2-weighted images in the coronal plane were obtained as described previously [19]. The cross section areas of the lateral ventricles and total brain were measured by an image analysis program (MetaMorph).

### Measurement of methylxanthines plasma level

To measure the plasma levels of caffeine and theophylline, a metabolite of caffeine, the rats were sacrificed around 1:00 PM, 3 days after the MRI. Blood was removed directly from the heart and was rapidly centrifuged, and the plasma collected. The procedure used for extraction and assay of the methylxanthines has been previously reported [20]. The HPLC analysis was performed as described by Kaplan *et al *[21].

### Animal preparation and measurement of CSF production

Animal preparation for measurement of CSF production and cerebral blood flow was performed as previously described [22]. The rectal temperature was kept at ~37°C. The arterial O_2 _tension was maintained at 100-120 mmHg by adjusting the inspired O_2 _content, and the arterial CO_2 _tension was maintained at 33-38 mmHg by adjusting the tidal volume and respiratory rate. Arterial blood pressure and pH were monitored as previously described [22]. The rate of CSF production was measured by the ventriculocisternal perfusion method [22]. The animals were mounted in a stereotaxic frame and a stainless steel cannula (27 gauge) was introduced into the lateral ventricle. Artificial CSF, containing Blue Dextran 2000 (Sigma-Aldrich, USA), at a concentration of 5 mg/ml, was infused through the cannula at a rate of 4 μl/ml, while intraventricular pressures were continuously monitored by means of T connectors inserted into the infusion lines. A stainless steel cannula (27 gauge) was inserted into the cisterna magna to enable CSF collection. CSF samples were collected at 10- min intervals and the concentration of Blue Dextran in the samples was determined colorimetrically by measuring the absorbance at 620 nm. The rate of CSF production (V_f_) in μl/ml was calculated from the dilution of Blue Dextran 2000 with the formula V_f _= V_i_(C_i_-C_o_)/C_o _where V_i _is the rate of infusion of the perfusate, and C_i _and C_o _are the concentrations of Blue Dextran in the inflow and outflow fluids, respectively. For each experiment, standard curves were prepared using known concentrations of Blue Dextran. To examine the acute effects of adenosine receptor signaling, on CSF production, the following drugs were injected intravenously after a 60-min ventriculocisternal perfusion. The choice of drugs and their doses were based on previous reports[3,23,25]: caffeine (10 mg/kg); [7-(2-phenylethyl)-5-amino-2-(2-furyl)-pyrazolo-4,3-e]-1,2,4-triazolol[1,5-c]pyrimidine (SCH58261, A_2A _receptor antagonist, Sigma-Aldrich, USA, 0.03 mg/kg); 2-p-(2-carboxy-ethyl)phenethylamino-5'-N-ethylcarboxamido-adenosine (CGS21680, A_2A _adenosine receptor agonist, Sigma-Aldrich, USA, 0.5 mg/kg); 8-cyclopenthyl-1,3-dipropylxanthine (DPCPX, A_1 _receptor antagonist, Sigma-Aldrich, USA, 0.1 mg/kg); N6-cyclopentyladenosine, (CPA, A_1 _adenosine receptor agonist, Sigma-Aldrich, USA, 0.1 mg/kg). These drugs were administered intravenously. To measure the respiratory rate, the up-and-down movement of the abdomen was counted for 1 min.

### Measurement of cerebral blood flow (CBF)

A craniectomy (5 mm in diameter, 2-4 mm lateral and 1-2 mm caudal to the bregma) was performed with extreme care over the middle cerebral artery territory. The dura was kept intact to prevent injury to the cortex. The field was frequently irrigated with physiological saline. To measure CBF, a 2.0 mHz pulsed wave Doppler transducer was attached over the temporal bone windows. In the resting state, the velocity from the middle cerebral artery was monitored using TCD equipment (Pioneer TC4040, Nicolet Vascular Doppler, USA). To measure cerebral perfusion, a laser Doppler perfusion imager (Moor Instrument, Devon, UK) was used as described previously [26].

### Immunohistochemistry

Immunohistochemistry was performed as previously described [27]. The animals were anesthetized with pentobarbital sodium (50 mg/kg) and sacrificed by intracardiac perfusion with 4% paraformaldehyde in 0.1 M phosphate buffer, pH 7.4. The brains were post-fixed for 4 h at 4°C with the same fixative and cryoprotected with 30% sucrose. The tissues were frozen using OCT compound embedding medium in dry ice powder. Coronal sections were cut (30 μm) using a cryostat (LEICA, CM3050, Germany) and processed for immunohistochemistry. Every sixth section, from the corpus striatum to the caudal hippocampal region, was immunostained. The sections were incubated in a blocking buffer (0.3% Triton X-100 and 10% goat or horse serum in PBS) for 1 h, and then incubated overnight with primary antibodies in an incubation buffer (0.1% Triton X-100, 1% goat or horse serum and 1% bovine serum albumin in PBS) at 4°C. The sections were then washed three times for 10 min each with PBS and incubated in HRP-conjugated secondary antibodies (1:200) for 2 h at room temperature. Then the DAB reaction was performed. The following primary antibodies were used: anti-Na^+^, K^+^-ATPase (1:500, Hybridoma Bank), anti-aquaporin 1 (AQP1) (1:500, Chemicon), anti-carbonic anhydrase II (CAII) (1:500, Megabase), anti- A_1 _adenosine receptor (1:200, Chemicon), and anti-A_2A _adenosine receptor (1:200, Millipore) antibodies.

### BrdU immunohistochemistry

The rats were injected intraperitoneally with BrdU (50 mg/Kg) every 2 h for 2 days to label the proliferating choroid epithelial cells. The rats were sacrificed 1 h after the final injection, and perfused with 4% paraformaldehyde in phosphate buffer (pH 7.4). The brain was removed as a tissue mass and fixed for an additional 6 hrs in the same fixative and embedded in paraffin. After cutting with a microtome, sections 4 μm thick were deparaffinized and hydrated using xylene and graded methanol. Deparaffinized and hydrated brain sections were pretreated with 50% formamide/280 mM NaCl/30 mM sodium citrate at 65°C for 2 h, and incubated in 2 M HCl at 37°C for 1 h. The remaining procedures were the same as described above for "immunohistochemistry".

### Western blot analysis

To remove the choroid plexus and corpus callosum from the brain tissues, we sliced the brains. Under the stereoscope, we dissected them out as purely as possible using a sharp scalpel. For the Western blotting, the tissues were homogenized in an ice-cold lysate buffer containing a cocktail of protease inhibitors (Complete, EDTA-free: Roche, Germany). After sonication, the samples were centrifuged at 12000 g for 20 min at 4°C, and then the supernatants were stored at -80°C. Forty μg of tissue lysates were subjected to 10% sodium dodecyl sulfate-polyacrylamide gel electrophoresis (SDS-PAGE). After transfer to nitrocellulose membranes (Amersham, Little Chalfont, UK), the membranes were probed with the following primary antibodies: anti-Na^+^, K^+^-ATPase (1:1,000, Hybridoma Bank), anti-aquaporin 1 (1:1,000, Chemicon), anti-carbonic anhydrase II (1:1,000, Megabase), anti- A_1 _adenosine receptor (1:1,000, Alpha Diagnostics), and anti-A_2A _adenosine receptor (1:1,000, Millipore) antibody. After incubating the blots for 1 h at 25°C with secondary antibodies conjugated with biotin, they were incubated with the ABC Elite complex (Vectastain ABC Elite Kit, Burlingame, USA) and visualized with the ECL Blotting Analysis System (Amersham, Little Chalfont, UK).

### Data Analysis

The data were presented as the means ± SEM. The differences between the mean values of two groups were evaluated using the Student's *t*-test (unpaired comparison). For comparison of more than three groups, we used a one-way analysis of variance (ANOVA) followed by Tukey's multiple comparison analysis. *P *values < 0.05 were considered statistically significant.

## Results

**Figure 1:**
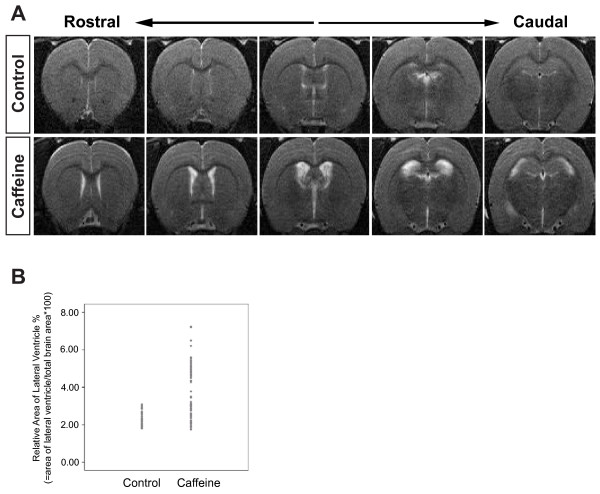
**Long-term consumption of caffeine can induce ventriculomegaly**. Caffeine (0.6 g/L) was added to the drinking water for 3 weeks (n = 72). **A**. Magnetic resonance images (T2-weighted) of rat brains in the coronal plane are presented anteroposteriorly from the striatum to the hippocampus. CSF in the ventricles appears white on the images. **B**. Measurement of relative cross section areas of the lateral ventricles by an image analysis program (MetaMorph). The relative area of the lateral ventricles was calculated as follows: area of lateral ventricles/total brain area*100.

Caffeine (0.3 or 0.6 g/L) was added to the drinking water for 3 weeks in the present study. This low dose of caffeine (0.3 g/L) has been shown to be representative of the standard daily human consumption [28]. Heavy coffee drinkers might ingest double this dose. While studying the effects of the long-term consumption of caffeine on brain function, we noted frequent enlargement of the ventricles in the caffeine-treated rats compared to the control rats. However, not all caffeine-treated rats developed ventriculomegaly. MRI was used to determine the frequency of ventriculomegaly in the caffeine-treated rats. A cross section, of the lateral ventricles, was measured using an image analysis program (MetaMorph, Figure 1). Significant enlargement of the lateral ventricles was noted in 40.0% or 41.7% of the caffeine-treated rats at a dose of 0.3 or 0.6 g/L respectively (n = 10 for 0.3 g/L, n = 72 for 0.6 g/L) (Figure 1). The average cross section area of the lateral ventricles in the caffeine-treated rats, receiving 0.3 or 0.6 g/L, had an increase of 2.0 to 2.2 fold in the ventricle size compared to the control rats. However, the difference between the two groups was not significant. To confirm the MRI findings, the brains were dissected and stained with hematoxylin and eosin (H&E). The H&E staining clearly showed a significant enlargement of the lateral ventricles (data not shown). Immunochemistry and Western blot analysis were performed on rats that had lateral ventricles two times larger than the control rats. In addition, 0.6 g/L of caffeine was added to the drinking water for long-term consumption experiments.

To determine why only 40% of the caffeine-treated rats had ventriculomegaly, we measured the plasma levels of caffeine after performing the MRI. The plasma levels of caffeine in the rats with ventriculomegaly was 3 times higher than in rats without ventriculomegaly (0.037 ± 0.008 versus 0.010 ± 0.006 μg/ml, *P *< 0.05, Table 1). The range of plasma caffeine concentrations observed in the rats with ventriculomegaly, was consistent with previous reports [14].

**Table 1:** Plasma concentrations of caffeine and its metabolite theophylline in caffeine-treated rats.

	Caffeine (μg/ml)	Theophylline (μg/ml)
Caffeine-treated rats with ventriculomegaly	0.037 ± 0.008	0.68 ± 0.089
Caffeine-treated rats without ventriculomegaly	0.010 ± 0.006	0.34 ± 0.174

Rats were treated with caffeine (0.6 g/L) for 3 weeks and were sacrificed 3 days after MRI imaging. Means ± SEM of 10 rats in each group

**Figure 2:**
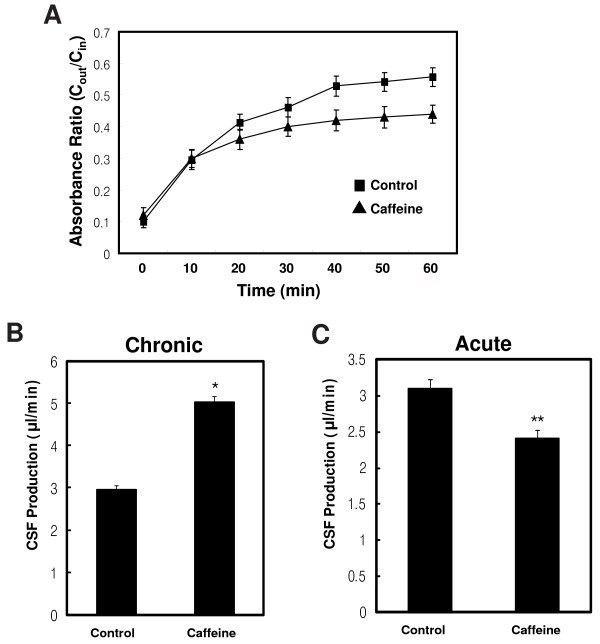
**The chronic administration of caffeine increased CSF production and acute administration decreased it**. **A**. The effect of chronic administration of caffeine on Blue Dextran recovery (C_ou_t/C_in_) from the CSF throughout a 60-min ventriculocisternal perfusion. The CSF was collected from the cisterna magna beginning immediately after the infusion. **B**. The effect of chronic administration of caffeine on the CSF production rate. **C**. The effect of acute administration of caffeine on the CSF production rate. Values are expressed as means ± SEM from 10 rats in each group. *, *P *< 0.01; **, *P *< 0.05 versus control rats

Ventriculomegaly can be caused by disturbances of CSF dynamics [29]. If the circulation pathway of the CSF is blocked, this usually leads to dilatation of the ventricles, since the production of fluid usually continues despite the obstruction. Another mechanism is overproduction of CSF. At first, we focused on the CSF pathway. When we analyzed MR images and paraffin sections of the brains, there was no gross physical obstruction in the CSF pathway identified (data not shown). Next, we measured the production of CSF using a ventriculo-cisternal perfusion technique. We found a significant increase in the production of CSF in the caffeine-treated rats compared to the control rats (5.02 ± 0.15 versus 2.95 ± 0.12 μl/min, *P *< 0.01, Figure 2).

The choroid plexus is the major site of CSF production [29]. The thin-walled vessels of the choroid plexus allow for passive diffusion of substances from the blood plasma into the extracellular space surrounding the choroid epithelial cells. Active transport of CSF is controlled by numerous transporters that mediate movement across the choroid epithelium. Na^+^, K^+^-ATPase establishes a sodium gradient across the choroid epithelial cells [30]. Other proteins including aquaporin I (AQP1) and carbonic anhydrase II also play an important role in CSF production.

**Figure 3:**
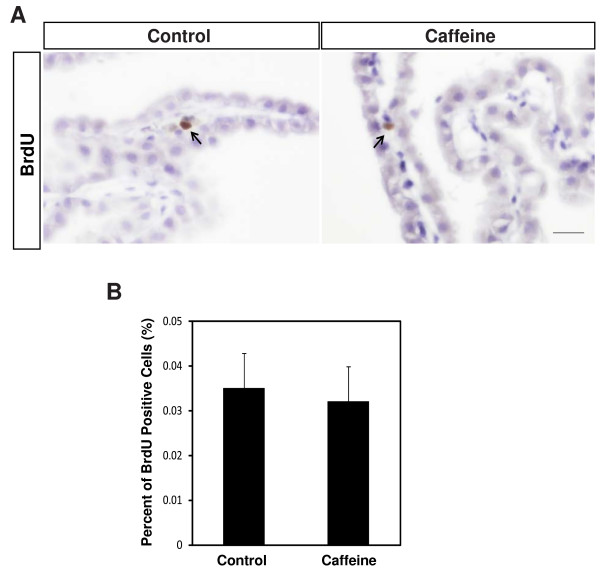
**No hyperplasia of choroid epithelial cells was detected in the caffeine-treated rats**. **A**. Representative photographs of BrdU immunohistochemistry in the choroid epithelial cells of the control and caffeine-treated rats (n = 4). BrdU incorporation into choroid epithelial cells was very rare. BrdU incorporation was indicated by arrows. Scale bar, 100 μm. **B**. Frequency of BrdU-positive cells in choroid epithelial cells. Values are expressed as the percentage of BrdU-positive cells (n = 4).

**Figure 4:**
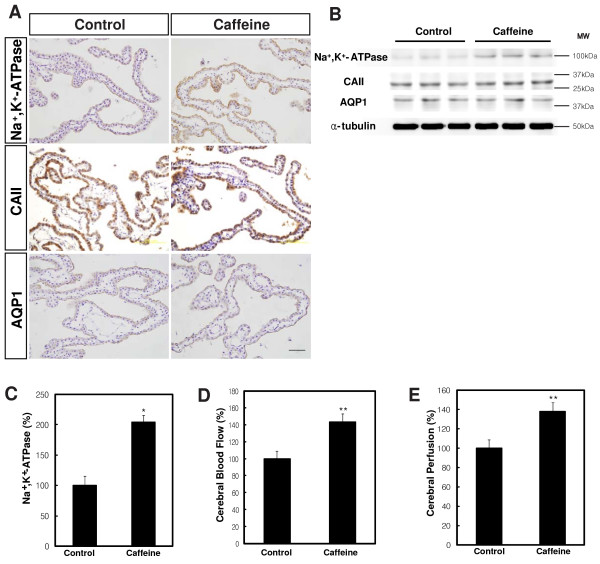
**Overproduction of CSF in caffeine-treated rats was associated with induction of Na^+^, K^+^-ATPase and increase in cerebral blood flow (CBF)**. **A**. Representative photographs of Na^+^, K^+^-ATPase, AQP1 and carbonic anhydrase II (CAII) immunohistochemistry in the choroid epithelial cells of the control and caffeine-treated rats (n = 4). Scale bar, 100 μm. **B**. Representative Western blots of Na^+^, K^+^-ATPase, AQP1 and CAII. **C**. Analysis of Western blots by image analyzer (LAS 3000, Fujifilm, Japan). α-tubulin was used as a control. **D**. CBF was increased in the caffeine-treated rats. **E**. Cerebral perfusion was increased in the caffeine-treated rats. Values are expressed as the percentage of control (n = 10). *, *P *< 0.01; **, *P *< 0.05 versus control rats

To determine the underlying mechanisms involved in CSF overproduction, in the caffeine-treated rats, we evaluated the choroid plexus for hyperplasia. No hyperplasia of the choroid plexus was found by H&E staining or BrdU experiments in the enlarged ventricles of the caffeine-treated rats (Figure 3). Next we examined the expression of proteins essential for CSF production including Na^+^, K^+^-ATPase, AQP1, and carbonic anhydrase II by immunohistochemistry and Western blotting. We noted a significant increase in the expression of Na^+^, K^+^-ATPase (204.2 ± 11.8% of control, *P *< 0.01) but not AQP1 or carbonic anhydrase II (CAII) in the choroid epithelial cells of the caffeine-treated rats compared to the control rats (Figure 4A-C). Because CBF can also affect the production of CSF [31], we examined CBF using ultrasound Doppler (Nicolet Vascular Doppler). We noted a significant increase in the CBF of the caffeine-treated rats compared to the control rats (143.3 ± 10.4% of control, *P *< 0.05, Figure 4D). The increase of CBF in the caffeine-treated rats was further supported by an increase in cerebral perfusion (138.5 ± 9.2% of control, *P *< 0.05, Figure 4E).

To exclude the possibility that different amounts of water uptake might have affected the analysis of CSF production, we measured the water uptake of one rat in a cage for 3 weeks (n = 10 each group). There was no difference in the water uptake of this rat compared to the other rats (see Additional file 1). Moreover, there was no significant difference in the rate of weight gain between the control rats and caffeine-treated rats during the experimental period (see Additional file 2).

**Figure 5:**
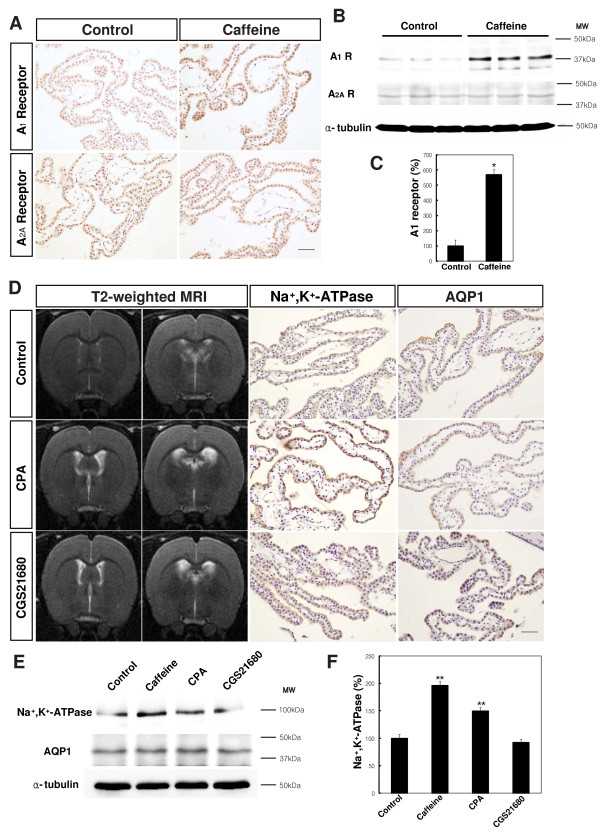
**The effect inversion of caffeine was associated with the induction of the A_1 _adenosine receptor after long-term caffeine treatment**. **A**. Representative photographs of adenosine A_1 _and A_2A _receptor immunohistochemistry in the choroid epithelial cells of the control and caffeine-treated rats (n = 4). Scale bar, 100 μm. **B**. Representative Western blots of the adenosine A_1 _and A_2 _receptors. **C**. Analysis of the Western blots using an image analyzer (LAS 3000, Fujifilm, Japan). α-tubulin was used as a control. **D**. Representative MR images (n = 27) and photographs of Na^+^, K^+^-ATPase and AQP1 immunohistochemistry of the choroid epithelial cells of the control, CPA-treated and CGS21680-treated rats (n = 4). Scale bar, 100 μm. An A_1 _agonist, CPA, and an A_2A _agonist, CGS21680, were associated with ventriculomegaly. CPA but not CGS21680 was correlated with an increased expression of Na^+^, K^+^-ATPase but not AQP1. **E**. Representative Western blots of Na^+^, K^+^-ATPase and AQP1. **F**. Analysis of the Western blots using an image analyzer (LAS 3000, Fujifilm, Japan). α-tubulin was used as a control. Values are expressed as a percentage of the control (n = 6). The results were analyzed by one-way ANOVA followed by Tukey's multiple comparison. *, *P *< 0.001; **, *P *< 0.05 versus control rats

Next, we examined the acute effects of caffeine on the production of CSF. Unexpectedly, the rats treated with caffeine just once, before measurement, showed a significantly reduced production of CSF, by 22.3% (n = 10, Figure 2C) compared to the control rats; this finding was contrary to the chronic effects of caffeine on CSF production. The acute treatment with caffeine had no significant effect on blood pressure and respiratory rate (see Additional file 3 &4). Because the "effect inversion" of caffeine might be caused by up-regulation of a receptor, we examined the expression of the adenosine receptors A_1 _and A_2A_, which have been identified as major targets of caffeine in the brain after chronic treatment with caffeine. The immunohistochemistry and Western blotting showed that the A_1_, but not the A_2A, _adenosine receptor, was increased in the choroid plexus of the caffeine-treated rats compared to the control rats (569.2 ± 32.3% of control, *P *< 0.001, Figure 5A &5B). This suggested that up-regulation of the A_1 _adenosine receptor was involved in the effect inversion of caffeine. To further confirm the involvement of the A_1 _receptor in the chronic effects of caffeine, we examined whether stimulation of the adenosine receptor was associated with the development of ventriculomegaly. Treatment with CPA, an agonist of the adenoinse A_1 _receptor, and CGS21680, an agonist of the adenosine A_2A _receptor, for 2 weeks caused ventriculomegaly in 37% of the agonist-treated rats (n = 27, Figure 5D). Interestingly, expression of Na^+^, K^+^-ATPase, but not AQP1, was increased in the CPA-treated (149.9 ± 7.2% of control, *P *< 0.05), but not the CGS21680-treated rats (Figure 5).

**Figure 6:**
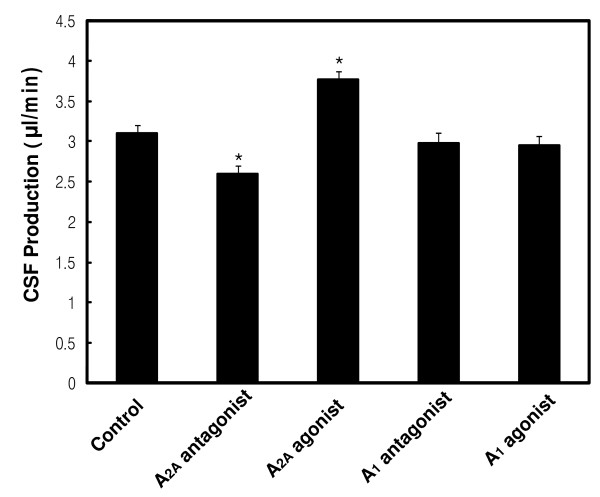
**Effects of acute administration of adenosine receptor agonists or antagonists on the CSF production rate**. Values are expressed as the means ± SEM of 10 rats in each group. *, *P *< 0.05 versus control rats.

Next, we examined the acute effects of A_1 _and A_2A _adenosine receptor agonists and antagonists on CSF production rate. The acute treatment with an A_2A _adenosine receptor agonist or antagonist increased or decreased CSF production rate by 21.4% or 16.1% respectively compared to the control rats (Figure 6). However, the acute effects of the A_1 _adenosine receptor agonist and antagonist, on CSF production, were not significant (Figure 6).

## Discussion

Numerous physical effects of caffeine consumption have been previously described. However, the effects of caffeine on brain structure and CSF production have not been reported on, to date. The results of the present study showed that long-term consumption of caffeine could cause ventriculomegaly, which appears to be mediated, in part, by increased production of CSF.

In addition to disturbances in CSF dynamics, reduction in white matter (periventricular leukomalacia) can cause secondary ventriculomegaly [32]. Periventricular leukomalacia can be caused by hypoxia, perinatal stress and sepsis. Previous studies have shown that hypoxia-induced ventriculomegaly is mediated by the A_1 _adenosine receptor, which is a major target molecule of caffeine in the brain [33,35]. Hypoxia-induced ventriculomegaly was not observed in mice that were deficient in the A_1 _adenosine receptor (*A1AR-/-*). By contrast, activation of the A_1 _adenosine receptor, during the first 2 postnatal weeks, leads to white matter injury. Moreover, ventriculomegaly was also observed in mice lacking the enzyme, adenosine deaminase, which degrades adenosine [33]. Although we cannot rule out the possibility of periventricular leukomalacia, caused by long-term consumption of caffeine, the radiologists did not find any of the signs of leukomalacia on the brain MRI of the caffeine-treated rats in the present study.

Different effects of short- and long-term caffeine exposure have been previously reported [11,17]. The effect inversion of caffeine was also observed in the present study. The acute treatment with caffeine reduced CSF production; however, chronic treatment increased CSF production. Three findings from the present study support up-regulation of the A_1 _adenosine receptor as associated with the observed effect inversion of caffeine. First, the expression of the A_1 _receptor was up-regulated in the caffeine-treated rats. Second, A_1 _agonist treatment was associated with the development of ventriculomegaly. Third, the expression of Na^+^, K^+^-ATPase was increased in both the caffeine-treated and the A_1 _agonist-treated rats. Although this continues to be debated, many previous reports have demonstrated up-regulation of the A_1 _adenosine receptor in caffeine-treated rats [14,36,39].

However, other mechanisms might also contribute to the effect inversion of caffeine. Conley et al. showed that the concentration of adenosine in the blood, after long-term consumption of caffeine, was increased more than tenfold [40]. This increase in the blood adenosine, after long-term consumption, might mediate the effect inversion of caffeine. However, the increase in the adenosine levels after long-term consumption of caffeine has never been replicated [41]. Another possible mechanism is the involvement of the A_2A _receptor. Because the A_2A _receptor plays a critical role in the regulation of the cerebral arteries [42,43], the increased CBF observed in the caffeine-treated rats might be mediated by the A_2A _receptor. Moreover, the A_2A _receptor agonist, CGS21680, also induced ventriculomegaly (Figure 5). However, in the present study, we did not find an increase in the expression of the A_2A _receptor in the caffeine-treated rats.

Recently, Yang et al showed that some aspects of the long-term consumption of caffeine can be replicated by genetic manipulation of the A_1 _and A_2A _adenosine receptors [41,44]. Caffeine (0.3 g/l in drinking water for 7-10 days) and the A_1_R-A_2A_R double heterozygote genotype increased locomotor activity and decreased the heart rate without significantly influencing body temperature. Despite the similarities in phenotype, the effects of long-term consumption of caffeine cannot be explained only by the blocking of the adenosine receptors. For example the reduction in heart rate after long-term consumption of caffeine is not due to the acute block of the adenosine receptors because acute caffeine administration does not produce a fall in the heart rate. Moreover, induction of Na^+^, K^+^-ATPase with long-term consumption of caffeine, in the present study, also cannot be explained by the blocking of or reduction in the adenosine receptors; this is because A_1 _adenosine receptor agonist treatment (Figure 5) and A_1 _adenosine receptor transgenic mice have been reported to have increased expression of Na^+^, K^+^-ATPase [45].

Several receptor signaling pathways have been implicated in the regulation of CSF production. Vasopressin and angiotensin II have been shown to decrease CSF production by epithelial effects and changes in blood supply. Involvement of the cAMP/PKA pathways and intracellular calcium, in these receptor signaling pathways, remains controversial. The results of the present study suggest that the adenosine receptor(s) may not directly regulate CSF production. Acute treatment with an A_1 _agonist or an A_1 _antagonist did not significantly change CSF production; these findings suggest that the A_1 _adenosine receptor may not regulate CSF production directly (Figure 6). Acute treatment with the A_2A _agonist or antagonist increased or decreased CSF production, respectively (Figure 6). However, the direct effects of the A_2A _adenosine receptor on epithelial cells could not be confirmed, because it regulates cerebral blood flow [42,43]. Therefore, the adenosine receptor appears to regulate CSF production by controlling the expression of Na^+^, K^+^-ATPase and cerebral blood flow. However, the acute and direct effects of the adenosine receptor on CSF production require further study.

Na^+^, K^+^-ATPase extrudes 3 Na^+ ^in exchange for 2 K^+ ^during the hydrolysis of 1 ATP molecule [30]. This enzyme is located in the luminal surface of the choroid epithelial cells. By contrast, it is located in the basolateral surface of all other transporting epithelia, both secretory and absorptive. The luminal Na^+ ^extrusion, by this enzyme, is the primary event and driving force of CSF production. Despite its critical role in CSF production, the regulation of its activity and expression has been poorly characterized. Na^+^, K^+^-ATPase is composed of α1, β1, β2 and phospholemman in the choroid plexus. In the present study, we showed that the A_1 _adenosine receptor regulates the expression of Na^+^, K^+^-ATPase in the choroid plexus; this finding is consistent with previous results showing increased expression of Na^+^, K^+^-ATPase in A_1 _adenosine receptor transgenic mice [45]. However, it has not been determined whether adenosine receptor signaling can regulate the activity of the enzyme directly. On the other hand, phospholemman has been suggested to be a target for phosphorylation-activated CSF production [30] Indeed, phospholemman is activated by PKA and has also been shown to be involved in the conduction of the anion current induced by cell swelling. However, PKA-dependent regulation of the Na^+^, K^+^-ATPase in the choroid plexus requires further study.

It is unclear why the blood caffeine levels of rats with ventriculomegaly were 3 times higher than in the rats without ventriculomegaly. There was no difference found in the daily water uptake and therefore the dose of caffeine was not significantly different between the controls and caffeine treated groups, consistent with a previous report [14]. Perhaps a difference in the metabolism of caffeine in the liver might explain these findings. The underlying mechanism needs to be investigated further.

## Conclusion

The results of this study show that the long-term consumption of caffeine can induce ventriculomegaly, which is mediated in part by increased production of CSF. Moreover, adenosine receptor signaling appears to regulate the production of CSF by controlling the expression of Na^+^, K^+^-ATPase and CBF.

## Abbreviations

AQP1: aquaporin 1; CBF: cerebral blood flow; CAII: carboinc anhydrase II; CGS21680: 2-p-(2-carboxy-ethyl)phenethylamino-5'-N-ethylcarboxamido-adenosine; CPA: N6-cyclopentyladenosine; DPCPX: 8-cyclopenthyl-1,3-dipropylxanthine; SCH58261: [7-(2-phenylethyl)-5-amino-2-(2-furyl)-pyrazolo-4,3-e]-1,2,4-triazolol[1,5-c]pyrimidine

## Authors' contributions

MEH found the enlargement of cerebral ventricles in caffeine-treated rats, participated in the design, measured CSF production and cerebral blood flow and drafted the manuscript. HJK participated in the design and coordination, carried out MRI and helped with the manuscript. YSL carried out immunohistochemistry and Western blot analysis. DHK carried out MRI and statistical analysis. JTC measured CSF production and carried out statistical analysis. CSP measured cerebral blood flow and cerebral perfusion and carried out statistical analysis. SY carried out immunohistochemistry. SYB measured methylxanthines plasma level and carried out statistical analysis. BSK and JBK participated in the experimental design and revision of the manuscript. SOO conceived of the study, participated in the design and coordination, and helped with the manuscript. All authors read and approved the final manuscript.

## Supplementary Material

Additional file 1 **There was no significant difference in the water uptake between the control and caffeine-treated groups**. Water uptake was examined everyday for 3 weeks. Caffeine (0.3 or 0.6 g/L) was added to the drinking water. Values are expressed as the means ± SEM of 10 rats in each group. Click here for file

Additional file 2 **There was no significant difference in weight gain between the control and caffeine-treated groups**. The weight gain was measured every week for 3 weeks. Caffeine (0.3 or 0.6 g/L) was added to the drinking water. Values are expressed as the means ± SEM of 8 rats in each group. Click here for file

Additional file 3 **Acute treatment with caffeine (10 mg/kg) did not cause a significant change in the mean arterial blood pressure (MABP)**. Caffeine was injected intravenously. Values are expressed as the means ± SEM of 8 rats in each group. Click here for file

Additional file 4 **Acute treatment with caffeine (10 mg/kg) did not cause a significant change in the respiratory rate**. The respiratory rate was counted for 1 min. The count was based on the up-and-down movement of the abdomen associated with the animal's breathing. Caffeine was injected intravenously. Values are expressed as the percentage of the control (n = 8). Click here for file

## References

[B1] Howell LL, Coffin VL, Spealman RD (1997). Behavioral and physiological effects of xanthines in nonhuman primates. Psychopharmacology.

[B2] Angelucci ME, Cesario C, Hiroi RH, Rosalen PL, Da Cunha C (2002). Effects of caffeine on learning and memory in rats tested in the Morris water maze. Brazilian journal of medical and biological research = Revista brasileira de pesquisas medicas e biologicas/Sociedade Brasileira de Biofisica.

[B3] Fredholm BB, Abbracchio MP, Burnstock G, Daly JW, Harden TK, Jacobson KA, Leff P, Williams M (1994). Nomenclature and classification of purinoceptors. Pharmacological reviews.

[B4] Fredholm BB, Battig K, Holmen J, Nehlig A, Zvartau EE (1999). Actions of caffeine in the brain with special reference to factors that contribute to its widespread use. Pharmacological reviews.

[B5] Field AS, Laurienti PJ, Yen YF, Burdette JH, Moody DM (2003). Dietary caffeine consumption and withdrawal: confounding variables in quantitative cerebral perfusion studies?. Radiology.

[B6] Nehlig A (1999). Are we dependent upon coffee and caffeine? A review on human and animal data. Neuroscience and biobehavioral reviews.

[B7] Zhao G, Messina E, Xu X, Ochoa M, Sun HL, Leung K, Shryock J, Belardinelli L, Hintze TH (2007). Caffeine attenuates the duration of coronary vasodilation and changes in hemodynamics induced by regadenoson (CVT-3146), a novel adenosine A2A receptor agonist. Journal of cardiovascular pharmacology.

[B8] El Yacoubi M, Ledent C, Parmentier M, Costentin J, Vaugeois JM (2003). Caffeine reduces hypnotic effects of alcohol through adenosine A2A receptor blockade. Neuropharmacology.

[B9] Huang ZL, Qu WM, Eguchi N, Chen JF, Schwarzschild MA, Fredholm BB, Urade Y, Hayaishi O (2005). Adenosine A2A, but not A1, receptors mediate the arousal effect of caffeine. Nature neuroscience.

[B10] Ledent C, Vaugeois JM, Schiffmann SN, Pedrazzini T, El Yacoubi M, Vanderhaeghen JJ, Costentin J, Heath JK, Vassart G, Parmentier M (1997). Aggressiveness, hypoalgesia and high blood pressure in mice lacking the adenosine A2a receptor. Nature.

[B11] Albertson TE, Joy RM, Stark LG (1983). Caffeine modification of kindled amygdaloid seizures. Pharmacol Biochem Behav.

[B12] Ault B, Olney MA, Joyner JL, Boyer CE, Notrica MA, Soroko FE, Wang CM (1987). Pro-convulsant actions of theophylline and caffeine in the hippocampus: implications for the management of temporal lobe epilepsy. Brain Res.

[B13] Georgiev V, Johansson B, Fredholm BB (1993). Long-term caffeine treatment leads to a decreased susceptibility to NMDA-induced clonic seizures in mice without changes in adenosine A1 receptor number. Brain Res.

[B14] Johansson B, Georgiev V, Kuosmanen T, Fredholm BB (1996). Long-term treatment with some methylxanthines decreases the susceptibility to bicuculline- and pentylenetetrazol-induced seizures in mice. Relationship to c-fos expression and receptor binding. Eur J Neurosci.

[B15] von Lubitz DK, Dambrosia JM, Kempski O, Redmond DJ (1988). Cyclohexyl adenosine protects against neuronal death following ischemia in the CA1 region of gerbil hippocampus. Stroke.

[B16] Rudolphi KA, Keil M, Fastbom J, Fredholm BB (1989). Ischaemic damage in gerbil hippocampus is reduced following upregulation of adenosine (A1) receptors by caffeine treatment. Neurosci Lett.

[B17] Sutherland GR, Peeling J, Lesiuk HJ, Brownstone RM, Rydzy M, Saunders JK, Geiger JD (1991). The effects of caffeine on ischemic neuronal injury as determined by magnetic resonance imaging and histopathology. Neuroscience.

[B18] Bayne K (1996). Revised Guide for the Care and Use of Laboratory Animals available. American Physiological Society. The Physiologist.

[B19] Mao X, Enno TL, Del Bigio MR (2006). Aquaporin 4 changes in rat brain with severe hydrocephalus. The European journal of neuroscience.

[B20] Fredholm BB, Herrera-Marschitz M, Jonzon B, Lindstrom K, Ungerstedt U (1983). On the mechanism by which methylxanthines enhance apomorphine-induced rotation behaviour in the rat. Pharmacology, biochemistry, and behavior.

[B21] Kaplan GB, Greenblatt DJ, Leduc BW, Thompson ML, Shader RI (1989). Relationship of plasma and brain concentrations of caffeine and metabolites to benzodiazepine receptor binding and locomotor activity. The Journal of pharmacology and experimental therapeutics.

[B22] Chodobski A, Szmydynger-Chodobska J, Johanson CE (1998). Vasopressin mediates the inhibitory effect of central angiotensin II on cerebrospinal fluid formation. European journal of pharmacology.

[B23] Akaiwa K, Akashi H, Harada H, Sakashita H, Hiromatsu S, Kano T, Aoyagi S (2006). Moderate cerebral venous congestion induces rapid cerebral protection via adenosine A1 receptor activation. Brain research.

[B24] Xiao D, Bastia E, Xu YH, Benn CL, Cha JH, Peterson TS, Chen JF, Schwarzschild MA (2006). Forebrain adenosine A2A receptors contribute to L-3,4-dihydroxyphenylalanine-induced dyskinesia in hemiparkinsonian mice. J Neurosci.

[B25] Poleszak E, Malec D (2003). Effects of adenosine receptor agonists and antagonists in amphetamine-induced conditioned place preference test in rats. Polish journal of pharmacology.

[B26] Riyamongkol P, Zhao W, Liu Y, Belayev L, Busto R, Ginsberg MD (2002). Automated registration of laser Doppler perfusion images by an adaptive correlation approach: application to focal cerebral ischemia in the rat. Journal of neuroscience methods.

[B27] Han ME, Park KH, Baek SY, Kim BS, Kim JB, Kim HJ, Oh SO (2007). Inhibitory effects of caffeine on hippocampal neurogenesis and function. Biochemical and biophysical research communications.

[B28] Barone JJ, Roberts HR (1996). Caffeine consumption. Food Chem Toxicol.

[B29] Meiniel A (2007). The secretory ependymal cells of the subcommissural organ: which role in hydrocephalus?. The international journal of biochemistry & cell biology.

[B30] Praetorius J (2007). Water and solute secretion by the choroid plexus. Pflugers Arch.

[B31] Faraci FM, Mayhan WG, Heistad DD (1990). Effect of vasopressin on production of cerebrospinal fluid: possible role of vasopressin (V1)-receptors. The American journal of physiology.

[B32] Volpe JJ (2001). Neurobiology of periventricular leukomalacia in the premature infant. Pediatric research.

[B33] Turner CP, Seli M, Ment L, Stewart W, Yan H, Johansson B, Fredholm BB, Blackburn M, Rivkees SA (2003). A1 adenosine receptors mediate hypoxia-induced ventriculomegaly. Proceedings of the National Academy of Sciences of the United States of America.

[B34] Wendler CC, Amatya S, McClaskey C, Ghatpande S, Fredholm BB, Rivkees SA (2007). A1 adenosine receptors play an essential role in protecting the embryo against hypoxia. Proceedings of the National Academy of Sciences of the United States of America.

[B35] Back SA, Craig A, Luo NL, Ren J, Akundi RS, Ribeiro I, Rivkees SA (2006). Protective effects of caffeine on chronic hypoxia-induced perinatal white matter injury. Annals of neurology.

[B36] Johansson B, Ahlberg S, Ploeg I van der, Brene S, Lindefors N, Persson H, Fredholm BB (1993). Effect of long term caffeine treatment on A1 and A2 adenosine receptor binding and on mRNA levels in rat brain. Naunyn-Schmiedeberg's archives of pharmacology.

[B37] Shi D, Nikodijevic O, Jacobson KA, Daly JW (1993). Chronic caffeine alters the density of adenosine, adrenergic, cholinergic, GABA, and serotonin receptors and calcium channels in mouse brain. Cellular and molecular neurobiology.

[B38] Shi D, Daly JW (1999). Chronic effects of xanthines on levels of central receptors in mice. Cellular and molecular neurobiology.

[B39] Shi D, Nikodijevic O, Jacobson KA, Daly JW (1994). Effects of chronic caffeine on adenosine, dopamine and acetylcholine systems in mice. Archives internationales de pharmacodynamie et de therapie.

[B40] Conlay LA, Conant JA, deBros F, Wurtman R (1997). Caffeine alters plasma adenosine levels. Nature.

[B41] Yang JN, Bjorklund O, Lindstrom-Tornqvist K, Lindgren E, Eriksson TM, Kahlstrom J, Chen JF, Schwarzschild MA, Tobler I, Fredholm BB (2009). Mice heterozygous for both A1 and A(2A) adenosine receptor genes show similarities to mice given long-term caffeine. J Appl Physiol.

[B42] Ngai AC, Winn HR (1993). Effects of adenosine and its analogues on isolated intracerebral arterioles. Extraluminal and intraluminal application. Circulation research.

[B43] Ngai AC, Coyne EF, Meno JR, West GA, Winn HR (2001). Receptor subtypes mediating adenosine-induced dilation of cerebral arterioles. American journal of physiology.

[B44] Yang JN, Chen JF, Fredholm BB (2009). Physiological roles of A1 and A2A adenosine receptors in regulating heart rate, body temperature, and locomotion as revealed using knockout mice and caffeine. American journal of physiology.

[B45] Lankford AR, Byford AM, Ashton KJ, French BA, Lee JK, Headrick JP, Matherne GP (2002). Gene expression profile of mouse myocardium with transgenic overexpression of A1 adenosine receptors. Physiological genomics.

