# Supplementary figures and images for: Regulation of cerebrospinal fluid production by caffeine consumption
Source: BMC Neurosci. 2009 Sep 3;10:110. doi: 10.1186/1471-2202-10-110 (PMC2744679; doi:10.1186/1471-2202-10-110)

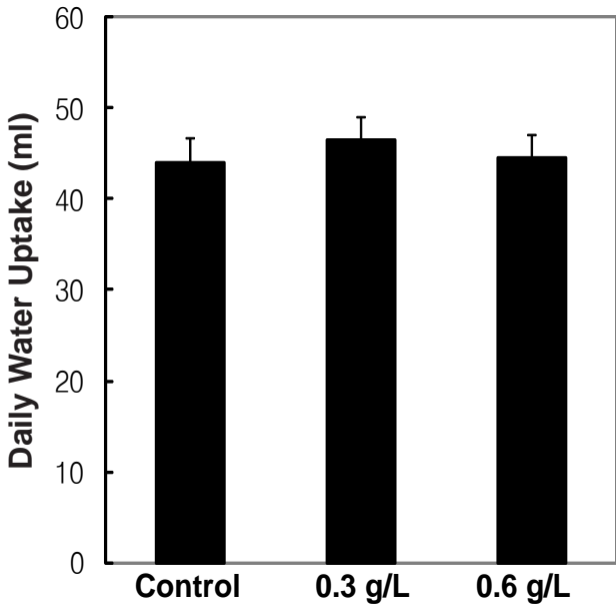

Supplement: Additional file 1 — There was no significant difference in the water uptake between the control and caffeine-treated groups. Water uptake was examined everyday for 3 weeks. Caffeine (0.3 or 0.6 g/L) was added to the drinking water. Values are expressed as the means ± SEM of 10 rats in each group. [file 1471-2202-10-110-S1.pdf]

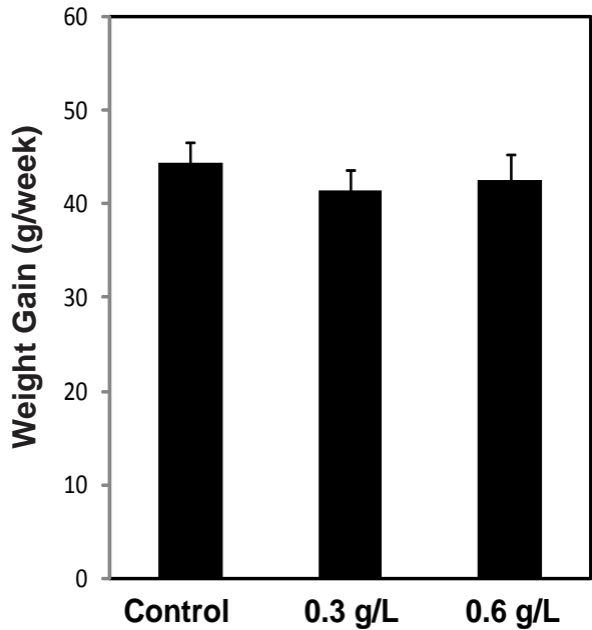

Supplement: Additional file 2 — There was no significant difference in weight gain between the control and caffeine-treated groups. The weight gain was measured every week for 3 weeks. Caffeine (0.3 or 0.6 g/L) was added to the drinking water. Values are expressed as the means ± SEM of 8 rats in each group. [file 1471-2202-10-110-S2.pdf]

**MABP (mmHg)**

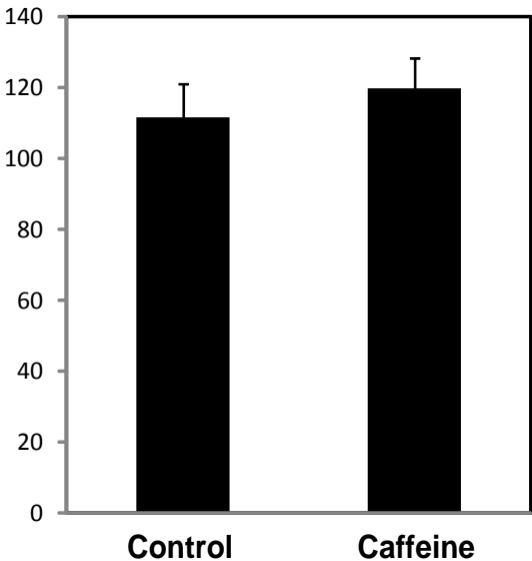

Supplement: Additional file 3 — Acute treatment with caffeine (10 mg/kg) did not cause a significant change in the mean arterial blood pressure (MABP). Caffeine was injected intravenously. Values are expressed as the means ± SEM of 8 rats in each group. [file 1471-2202-10-110-S3.pdf]

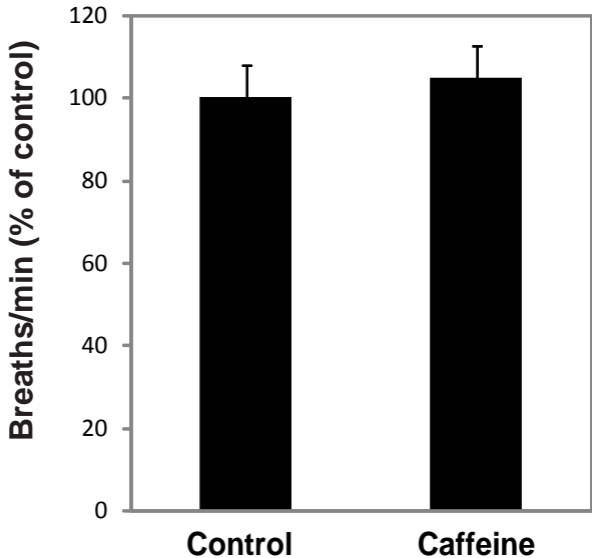

Supplement: Additional file 4 — Acute treatment with caffeine (10 mg/kg) did not cause a significant change in the respiratory rate. The respiratory rate was counted for 1 min. The count was based on the up-and-down movement of the abdomen associated with the animal's breathing. Caffeine was injected intravenously. Values are expressed as the percentage of the control (n = 8). [file 1471-2202-10-110-S4.pdf]
